# Fabrication of a modafinil co-crystal for enhanced dissolution, anti-depressant potential and density functional theory studies

**DOI:** 10.1039/d6ra05176f

**Published:** 2026-07-27

**Authors:** Zakir Hussain, Muhammad Naveed Umar, Adnan Khan, Haseena, Syed Wadood Ali Shah, Mehrin Ghias, Muhammad Zahoor, Amal Alotaibi

**Affiliations:** a Department of Chemistry, University of Malakand Chakdara 18800 Pakistan zakirhussain@uom.edu.pk m.naveedumar@uom.edu.pk adnankhan4754047@gmail.com haseenafarid5@gmail.com; b Department of Pharmacy, University of Malakand Chakdara 18800 Khyber Pakhtunkhwa Pakistan phramacistsyed@gmail.com mehrin.ghias19@gmail.com; c Department of Biochemistry, University of Malakand Dir Lower Chakdara KPK 18800 Pakistan mohammadzahoorus@yahoo.com; d Department of Basic Science, College of Medicine, Princess Nourah bint Abdulrahman University Riyadh 11671 Saudi Arabia amaalotaibi@pnu.edu.sa

## Abstract

In the current study, a modafinil (MO)-based co-crystal of MO-BA with benzoic acid (BA) as the co-former was prepared. The MO-BA formation was confirmed experimentally by FT-IR spectroscopy, DSC, and PXRD and elucidated theoretically *via* density functional theory (DFT). The broadening, shifting and attenuation of the –OH, –NH, –C=O and –S=O peaks in the FT-IR spectra confirm the synthesis of the MO-BA co-crystal. The appearance of endothermic peaks at different temperatures for MO-BA (112 °C) compared with MO (166 °C) and BA (122 °C) indicated the effective formation of MO-BA. Similarly, the diffractogram of MO-BA exhibited peaks with different 2*θ* values from those of MO and BA in terms of position as well as intensity. These fluctuations in diffraction patterns support the formation of a new crystalline phase. HPLC analysis showed that MO and BA combine in a 1 : 1 ratio to form MO-BA. QTAIM analysis revealed the presence of strong hydrogen bonding and van der Waals interactions, as evidenced at bond critical points based on Laplacian density, electron density and total energy density. The *in vitro* dissolution profiles of the MO-BA co-crystal and raw MO were found to be 51.75% ± 1.55% and 31.42% ± 1.48% in buffer solutions of pH 6.8 and 59% ± 1.20% and 37.61% ± 1.85% in acidic HCl medium of pH 1.3, respectively. The *in vivo* anti-depressant potentials of raw MO and MO-BA were evaluated by the forced swim test (FST) and tail-suspension test (TST). The observed immobility responses of MO and MO-BA at a dose of 30 mg kg^−1^ were 95.83 ± 1.13 and 89.66 ± 1.78 s in the FST and 119.16 ± 1.42 and 114.66 ± 1.58 s in the TST, respectively. This study provides insights into the effective formation of the MO-BA co-crystal with enhanced dissolution and improved anti-depressant potential compared with pristine MO, offering excellent bioavailability.

## Abstract 2

In the current study, a modafinil (MO)-based co-crystal of MO-BA with benzoic acid (BA) as the co-former was prepared.

## Graphical abstract

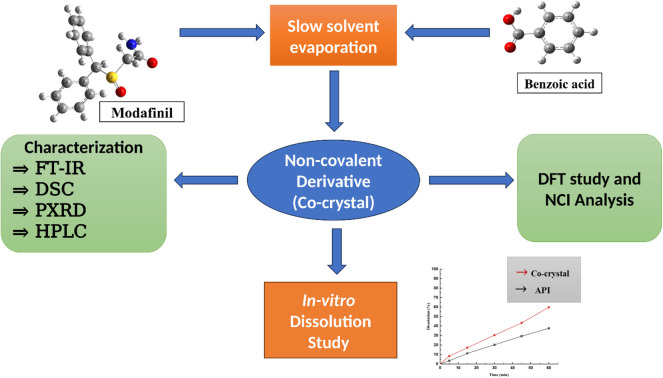

## 1 Introduction

Co-crystals of an active pharmaceutical ingredient (API) are formed by incorporating one or more co-former molecule(s) into its matrix through non-covalent interactions (NCIs).^1^ Hydrogen bonding, ionic interactions, π–π interactions, *etc.* are the NCIs involved in the formation of non-covalent derivatives (NCDs).^2^ Salts and co-crystals are two important NCDs that offer a unique way to improve key physicochemical properties without changing the chemical nature of the APIs.^3^ Solubility, dissolution rate, bioavailability, melting point, stability, tabletability, taste and other properties can be changed during salt formation and co-crystallization.^4–6^ The Δp*K*_a_ values of the acid and base generally determine whether the NCD would be a salt or a co-crystal. When Δp*K*_a_ (Δp*K*_a_ = p*K*_a_base__ − p*K*_a_acid__) >3, salt formation is favored, while Δp*K*_a_ < 0 favors co-crystal formation. The range (0 < Δp*K*_a_ < 3) is uncertain and can yield either salts or co-crystals depending upon the intermolecular interactions.^7^

The co-crystal of modafinil with nicotinic acid was synthesized with the aim of enhancing the solubility and bio-availability compared with raw modafinil. It was found that the aqueous solubility and *in vivo* bio-availability of the co-crystals of modafinil and nicotinic acid were about 5.96 and 1.88 times higher than that of raw modafinil, respectively.^8^ In our previous study, we reported the co-crystals of modafinil with tartaric acid and adipic acid.^9^ However, no such attempts have been made for the enhancement of modafinil solubility or co-crystal synthesis. Co-crystals can also enhance patient compliance due to enhanced formulation taste. Increased solubility and rapid release of the API were also observed for atorvastatin calcium co-crystals with nicotinamide and citric acid.^10^ For the preparation of co-crystals, different methods have been described in the literature, such as solid-state and solution-based methods.^4^ Solution-based methods consist of slow solvent evaporation, slurry formation, cooling crystallization, anti-solvent methods and reaction co-crystallization. Among these techniques, slow solvent evaporation is a unique method that is used for preparing of single larger crystals in which the API and co-former are present in equal ratios.^11,12^

Modafinil (MO) is a Biopharmaceutical Classification System (BCS) II drug with the molecular formula C_15_H_15_NO_2_S. The chemical name of modafinil is 2-(diphenylmethanesulfinyl)acetamide, and the compound is used to manage narcolepsy and depression. It is also an alertness agent and was approved by the FDA as a Schedule IV agent for treating sleep disorders.^13,14^ Patients suffering from narcolepsy and depression have low levels of hypocretin. MO binds to the dopamine transporter, due to which the level of dopamine increases in the human brain and the level of hypocretin also increases indirectly, thereby lowering the severity of narcolepsy and depression.^15,16^ The amide group present in MO has the potential to participate in NCIs with co-formers. MO is poorly soluble in water and thus absorbed weakly in the intestinal tract, resulting in low bio-availability.^8,17,18^ The absorption of MO in the intestinal tract will be increased and excellent bioavailability may be achieved if its solubility issue is resolved. Therefore, the solubility and dissolution of MO must be enhanced for better absorption and bio-availability to attain good pharmacological effects. Benzoic acid (BA) is an aromatic carboxylic acid that is used as a co-former with fenofibrate for better aqueous solubility, dissolution rate, and other pharmacological parameters.^19^ In a previous study, the co-crystal of theophylline with benzoic acid was reported to have enhanced solubility and bioavailability.^20^ No co-crystal of modafinil with benzoic acid has been reported. In the context of supramolecular chemistry, benzoic acid contains a carboxylic group, which enables it to act as both a hydrogen-bond donor and a hydrogen-bond acceptor. Due to these capabilities, it can yield predictable heterosynthons with the amide and sulfinyl groups of modafinil.^21^ As compared to citric acid, succinic acid, and fumaric acid, which are multifunctional co-formers,^22^ benzoic acid is simpler and more directional in terms of hydrogen bonding and favors the synthesis of well-defined co-crystals.^23^ Derivatives of benzoic acid, like *p*-aminobenzoic acid, hydroxybenzoic acid, and fluorinated benzoic acid, are also used as co-formers with APIs; *e.g.*, chlordiazepoxide,^24^ ketoconazole, and naftopidil, respectively.^25,26^ The anti-depressant potential of MO was evaluated by J. Mahmoudi *et al.* in rats by the tail suspension test, and it was reported that it produced an antidepressant effect.^27^

The current study aims to synthesize the co-crystal of MO with BA for enhancing its dissolution/bio-availability and anti-depressant potential. To confirm the feasibility of the co-crystals and to determine the nature and type of the NCIs, DFT simulations and quantum theory of atoms-in-molecules (QTAIM) analyses were performed. This study presents experimental as well as theoretical findings on the modafinil-benzoic acid co-crystal and demonstrates its better dissolution and *in vivo* anti-depressant efficacy. To the best of our knowledge, no such study has been reported previously.

## 2 Experimental and theoretical details

### 2.1 Materials

MO was provided by Proventus Life Sciences Chemicals. Ethanol, benzoic acid, HCl, methanol and KH_2_PO_4_ were bought from Sigma-Aldrich and used without any refinement.

### 2.2 Synthesis of co-crystal

**Scheme 1:**
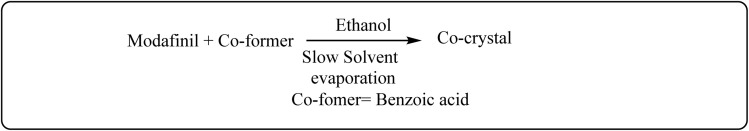
Synthesis of the co-crystal of modafinil.

The MO-based MO-BA co-crystal was synthesized by the slow solvent evaporation process. In this technique, equimolar solutions of MO and co-former BA were prepared in ethanol in separate vessels. The solutions of MO and BA were then mixed in a vessel. The vessel containing the solution of MO and BA was then kept in the dark, and the solvent ethanol was allowed to evaporate slowly. Upon complete evaporation of the solvent, crystals appeared, which were detached carefully, dried, and kept in the dark for further assessment (Scheme 1).^28^

### 2.3 Characterization techniques

FTIR analysis of the samples (MO, BA and MO-BA) was performed using an FTIR spectrophotometer (PerkinElmer spectrum) over a scanning range of 4000–400 cm^−1^. DSC analysis of the samples was conducted with a simultaneous thermal analyzer (STA-8000; PerkinElmer, USA) with a heating range of 25–250 °C at a rate of 10 °C min^−1^, under a continuous flow of an inert nitrogen atmosphere.^24^ Moreover, the co-crystal (MO-BA) and starting materials (MO and BA) were screened to collect X-ray diffraction patterns using the PXRD technique. The diffractometer used was an AXRD-LPD Proto USA with a 2*θ* scanning range of 6°–50° and counts for one sec step^−1^.

### 2.4 DFT studies and NCI analysis

To evaluate the nature of the non-covalent interactive forces between MO and co-former BA and to predict the stability of the co-crystal, quantum chemical calculations were performed by applying the Dmol^3^ code, implemented in Material Studio.^29,30^ The Perdew–Burke–Ernzerhof (PBE) functional of the generalized-gradient approximation (GGA) was used^31^ to optimize the geometries of the compounds with the basis set double-numerical-plus-polarization (DNP) and Grimme's D3 dispersion correction.^32^ For all structural relaxation, thermal smearing of 0.005 Ha, a basis set cutoff of 4.6 Å, and convergence thresholds of 10^−5^ Ha for energy, 0.005 Å for displacement, and 0.001 Ha Å^−1^ for force were implemented. Moreover, the implicit solvation effects were introduced using the conductor-like screening model (COSMO) with water as the solvent (*ε* = 78.5).^33^ The formation energy (*E*_form_) of the MO-BA co-crystal was calculated using the following equation:

1 *E*_form_ = *E*_co-crystal_ − (*E*_MO_ + *E*_co-former_),

where *E*_co-crystal_, *E*_MO_, and *E*_co-former_ are the energies of optimized MO-BA, MO, and BA (co-former), respectively.

NCIs responsible for the stability of the co-crystal were studied using the QTAIM^34^ framework, and reduced density gradient (RDG) analysis^35^ was used to study the non-covalent interactions (NCIs) in the co-crystal *via* the Multiwfn and visual molecular dynamics (VMD) programs. The Gaussian 09 package was applied to generate wavefunction files.^36,37^

### 2.5 Saturation solubility and HPLC analysis

A supersaturated solution of MO was prepared in a reciprocating shaker water bath by adding an excess amount of MO to 50 mL of distilled water and allowing it to dissolve by stirring at room temperature. The supersaturated solution of MO was then filtered and its absorbance was recorded with a UV-visible spectrophotometer. The process was performed in triplicate and the mean values were noted. Using the same procedure, the absorbance of the MO-BA solution was recorded with a UV-visible spectrophotometer.^38^

A previously reported method was used to determine the concentration of MO in MO-BA. An Agilent-1260 (Shimadzu, Japan) with a PDA detector was used, and the analysis was performed using a ZORABEX Eclipse plus C18 reversed-phase column. The mobile phase used was methanol : phosphate buffer (9 : 1 v/v) with a flow rate of 1.2 mL min^−1^, the PDA detector temperature was adjusted to 40 °C, and the injection volume was 50 µL.^38,39^ The concentration of MO was measured by running MO-BA solution (1 mg mL^−1^) in the mobile phase.

### 2.6 *In vitro* dissolution study

The paddle method was applied to determine the *in vitro* dissolution potential of MO and MO-BA, for which a USP apparatus type-2 was used. The samples of raw MO and MO-BA were first ground into a fine powder. 900 mL of phosphate buffer with pH 6.8 was taken in each vessel of the paddle apparatus. 30 mg of each sample (MO or MO-BA) were added to separate vessels. The rotation speed of the paddle was adjusted to 70 rpm and the temperature was set at 37 °C. To determine the comparative drug release from raw MO and MO-BA, 5 mL from each vessel were withdrawn after regular intervals (*i.e.*, 5, 15, 30, 45, and 60 min). 5 mL of buffer solution (dissolution medium) was added after each withdrawal to the vessels to keep the volume at 900 mL. The drug release from the sample was determined by using a UV-visible spectrometer.^40,41^ The same technique was adopted to determine the drug release from raw MO and MO-BA in HCl solution at pH 1.3.

### 2.7 *In vivo* anti-depressant activity of raw MO and its co-crystal

The anti-depressant potential of raw MO and MO-BA was determined by subjecting animals to forced swimming tests and tail-suspension tests after treatment.

#### 2.7.1 Forced swim test (FST)

Mice were randomly categorized into three groups (negative control group, standard group and test groups), with each group having six mice. One group was taken as a negative control to which only the vehicle Tween 80 was administered. Test groups were administered with 30 mg kg^−1^ of MO or MO-BA separately and a standard group was given imipramine. After 30 min of dose, the mice were forced to swim in a cylinder filled with water. The immobility responses were noted by using a stopwatch for 6 min.^42^

#### 2.7.2 Tail-suspension test (TST)

Mice were randomly categorized into groups, with each group having 6 mice. One group was taken as the negative control, to which only the vehicle Tween 80 was administered. Test groups were administered with 30 mg kg^−1^ of MO or MO-BA separately. The standard group was given imipramine. After 30 min of dose, mice were suspended from a height by their tail. The immobility responses were noted by using a stopwatch for 6 min.^42^

## 3 Results and discussion

### 3.1 Δp*K*_a_ values and melting points of MO and MO-BA

The Δp*K*_a_ values (Δp*K*_a_ = p*K*_a_base__ − p*K*_a_acid__) of bases and acids give preliminary information on the formation of a co-crystal or salt. Generally, a co-crystal is formed when Δp*K*_a_ ≤ 0 and a salt is formed when Δp*K*_a_ > 3. The formation of MO-BA as a co-crystal is consistent with the Δp*K*_a_ rules (also called the rule of 3) and Δp*K*_a_ < 0. The Δp*K*_a_ value of MO and BA is −8.6 and was calculated from the formula: Δp*K*_a_ = p*K*_a_base__ − p*K*_a_acid__. The p*K*_a_ of MO is −4.4, which refers to the conjugate acid of MO, while the p*K*_a_ of BA is 4.2; by using this formula, the Δp*K*_a_ was found to be −8.6. The melting points (mp) of MO, BA and MO-BA were determined and are given in Table 1. The melting point of MO-BA differs from those of the starting materials, which provided primary evidence of its successful formation.

**Table 1:** Experimental results of MO, BA and MO-BA

Exp. results	Modafinil (MO)	Benzoic acid (BA)	MO-BA
mp (°C)	166–168	120–122	110–112
N–H (cm^−1^)	3298 (*asym*)	—	3378 (*asym*)
	3163 (*sym*)		3189 (*sym*)
C=O (cm^−1^)	1684	1677	1686
S=O (cm^−1^)	1470	—	1491
Endothermic peak (°C)	166	—	112
2*θ* (°) high intensity peaks	9.09°, 15.77°, 18.19°, 19.32°, 20.50°, 24.57°, 27.28° and 33.10°	14.28°, 18.24°, 20.35°, 23.38°, 28.52°, 33.82° and 44.61°	13.77°, 16.03°, 19.12°, 22.35°, 25.13°, 29.91°, 35.36°, 36.54° and 39.73°

### 3.2 FT-IR spectroscopy

Non-covalent interactions (NCIs), *e.g.* hydrogen bonding, are involved in the formation of co-crystals.^43^ NCIs result in changes in the vibrational frequencies; therefore, vibrational (FT-IR) spectroscopy is an excellent tool to study these interactions in co-crystals. In the formation of co-crystals, hydrogen bonding is involved.^44^ Thus, the detection of hydrogen bonding would signify the formation of co-crystals.

**Fig. 1:**
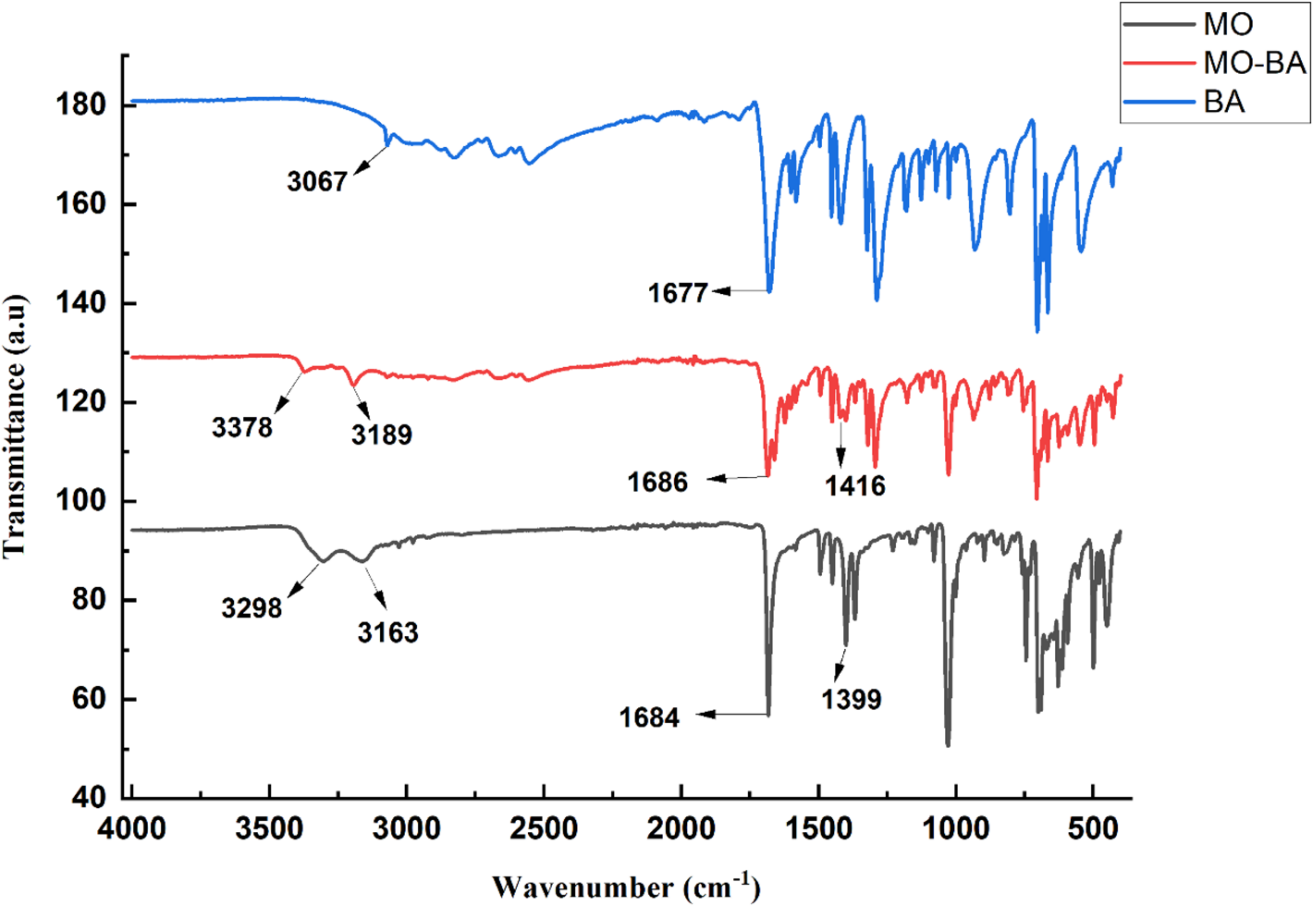
FTIR spectra of MO, MO-BA and BA (MO at 3298 = asymmetric –NH stretching and 3163 cm^−1^ = symmetric –NH stretching, BA at 2900–2500 cm^−1^ = broad peak due to –COOH and 1677 cm^−1^ = carbonyl group, and MO-BA at 3378 cm^−1^ = asymmetric –NH stretching and 3189 cm^−1^ = symmetric –NH stretching).

The FTIR spectra of MO, BA and MO-BA are displayed in Fig. 1. MO exhibited characteristic bands at 3298, 3163, 1684 and 1399 cm^−1^. The broad bands at 3298 and 3163 cm^−1^ are assigned to the symmetric and anti-symmetric stretching vibrations of the N–H bond, respectively, whereas the peaks at 1684 cm^−1^ and 1399 cm^−1^ are attributed to the stretching vibrations of the C=O and S=O groups, respectively. In addition, peaks around 3050 and 2900 cm^−1^ correspond to the aromatic–H and C–H vibrations, respectively. However, BA (blue curve, Fig. 1) showed broad bands at 2900–2500 cm^−1^ due to the carboxyl group, while the peak at 3067 cm^−1^ is attributed to Ar–H. The peaks at 1677 cm^−1^ and near 1500 cm^−1^ are attributed to the carbonyl group and aromatic ring, respectively.

In the case of the MO-BA co-crystal (red curve, Fig. 1), the pristine MO bands due to the –NH group (3298 and 3163 cm^−1^) were shifted to higher wavenumbers, *i.e.*, 3378 and 3189 cm^−1^ in the MO-BA co-crystal. Changes in the S=O and C=O wavenumbers were also observed. Hence, attenuation, augmentation, and overall shifts of the –OH, –NH, –S=O and C=O peaks suggested the establishment of H-bonding between the MO and BA, driving the formation of the co-crystal.

### 3.3 Differential scanning calorimetry (DSC)

**Fig. 2:**
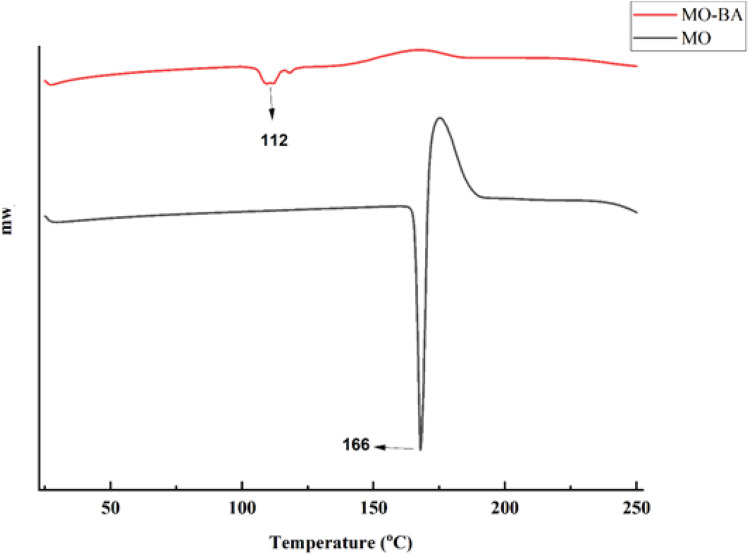
DSC curves of MO and MO-BA (endothermic peaks of MO and MO-BA at 166 °C and 112 °C, respectively).

To confirm the formation of the co-crystal, DSC analysis of MO and MO-BA was performed. Fig. 2 gives information on the thermal behavior of MO and MO-BA. An endothermic peak of MO was observed at 166 °C, while MO-BA exhibited an endothermic peak at 112 °C, and the melting temperature of BA is 122 °C.^20^ These peaks provide information about the formation of the MO-BA co-crystal. The shifts in the endothermic peaks of the co-crystal result from the strong interactions between the API (MO) and co-former (BA). The melting temperature of the co-crystal is less than those of both MO and BA; therefore, the DSC results support the formation of the co-crystal. Moreover, the difference in the melting points indicates that various physical interactions develop between the components (MO and BA) of the co-crystal (MO-BA). Previous studies reported that 39% co-crystals have melting points lower than both the API and co-former.^45,46^ The melting temperature of the co-crystal (MO-BA) in our study was observed to be less than those of the API (modafinil) and the co-former BA.

### 3.4 PXRD analysis

**Fig. 3:**
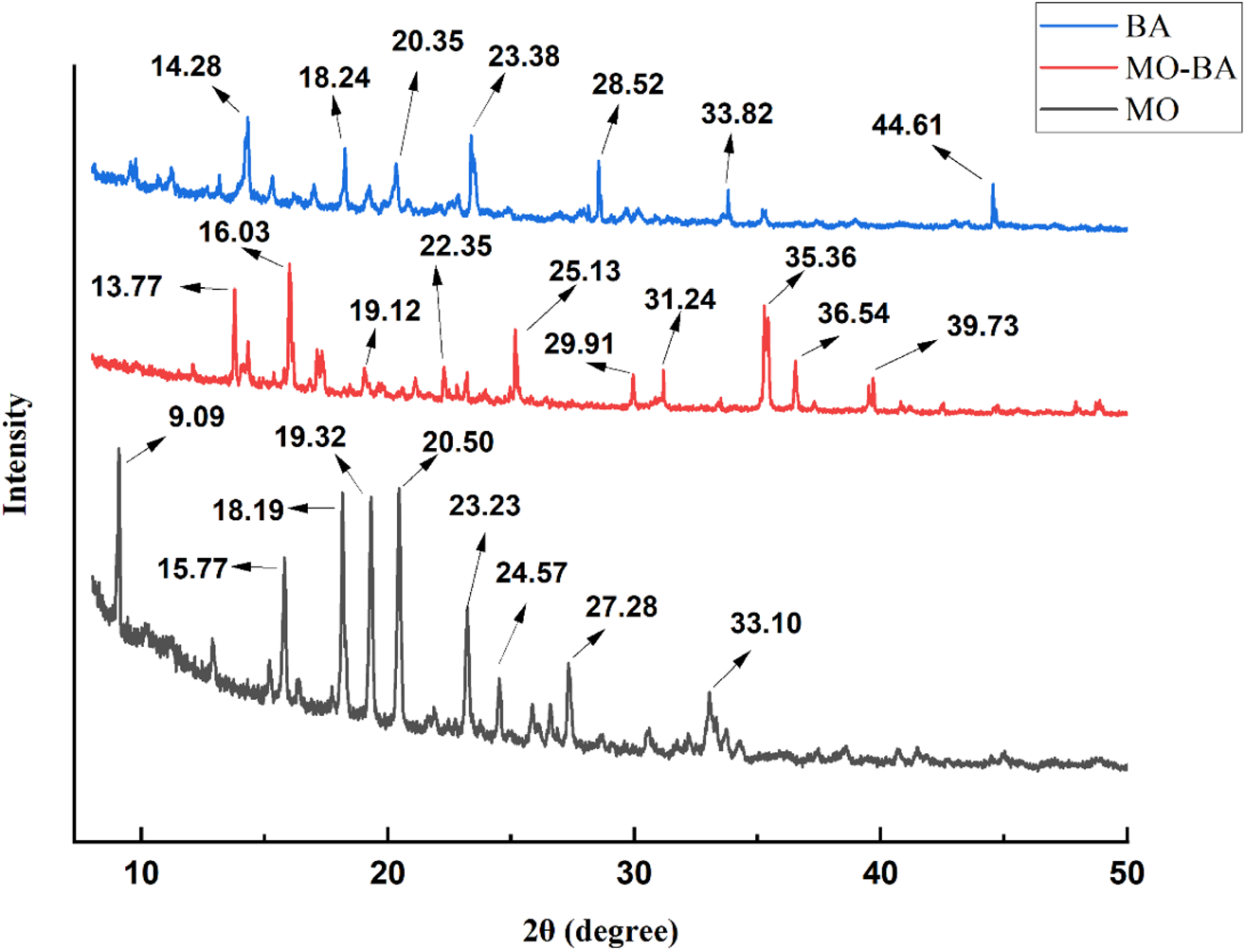
Powder XRD patterns of MO, MO-BA and BA.

PXRD is a useful technique that is used for the determination of crystallinity and new solid-phase identification.^47^ The diffractograms of MO, BA and MO-BA are shown in Fig. 3. MO (black pattern) exhibited high intensity peaks at 2*θ* values of 9.09°, 15.77°, 18.19°, 19.32°, 20.50°, 23.23°, 24.57°, 27.28° and 33.10°, while the BA diffractogram (blue pattern) showed high intensity peaks at 14.28°,18.24°, 20.35°, 23.38°, 28.52°, 33.82° and 44.61°. Similarly, MO-BA (red pattern) exhibited high-intensity peaks at 2*θ* values of 13.77°, 16.03°, 19.12°, 22.35°, 25.13°, 29.91°, 35.36°, 36.54° and 39.73°, which are not found in the diffractograms of the starting materials. Further, the diffraction patterns of MO-BA are completely different from those of MO and BA in terms of position as well as intensity. Previous studies described that such types of fluctuations in peak strength and position are usually brought about by co-crystal formation.^31,37^ This suggests that a new crystalline phase is formed, supporting the formation of the MO-BA co-crystal.

### 3.5 DFT study

**Fig. 4:**
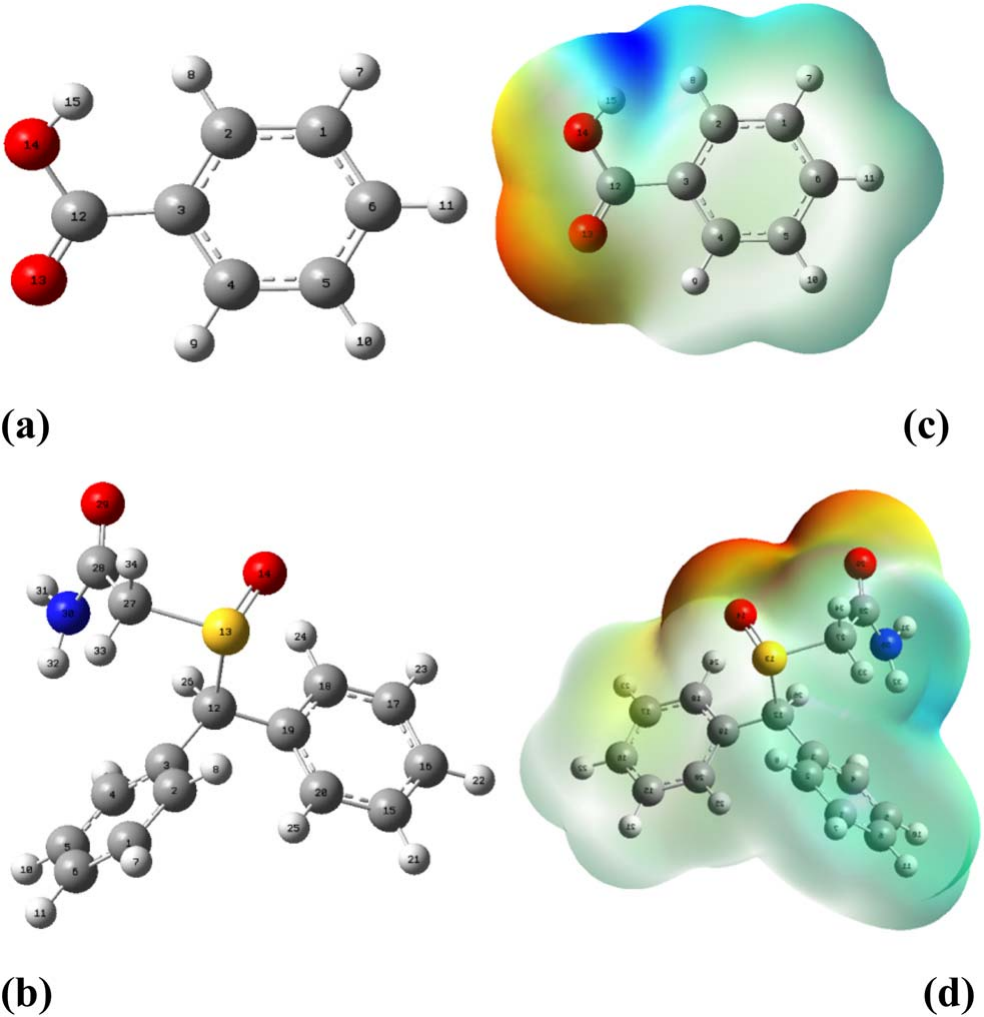
Optimized geometries of (a) BA, (b) MO and (c and d) their analogous MEP maps.

**Fig. 5:**
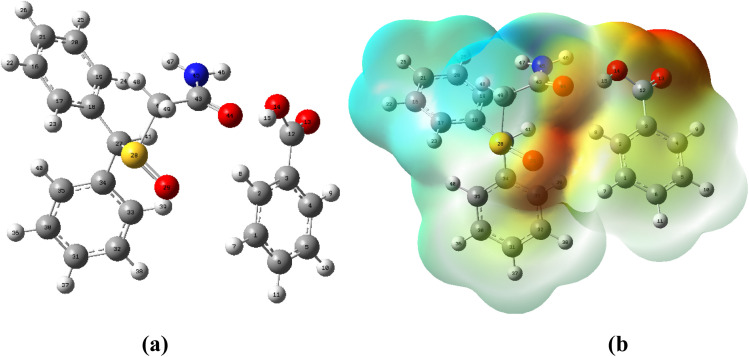
Optimized geometries of the MO-BA co-crystal (a) and its corresponding MEP map (b).

The optimized structures of MO, BA and their molecular electrostatic potential (MEP) maps are shown in Fig. 4. The MEP maps contain red and blue patches representing the electron-rich (electron donor) and electron-deficient (electron acceptor) sites. The carbonyl groups are encapsulated by red regions as they are electron-rich, while the acidic hydrogens are represented by blue regions. The optimized structure of the MO-BA co-crystal is obtained when the electron-rich and electron-deficient sites of the co-former (BA) and MO interact, as represented in Fig. 5. The optimized structure of MO-BA showed that BA interacts strongly with MO to form the co-crystal through intermolecular hydrogen bonding (MO) C=O⋯H–O–C=O (BA) and (MO) N–H⋯OH–C=O (BA) with bond distances of 1.73 and 2.06 Å, respectively. The *E*_form_ value for the MO-BA co-crystal is −0.71 eV.

**Fig. 6:**
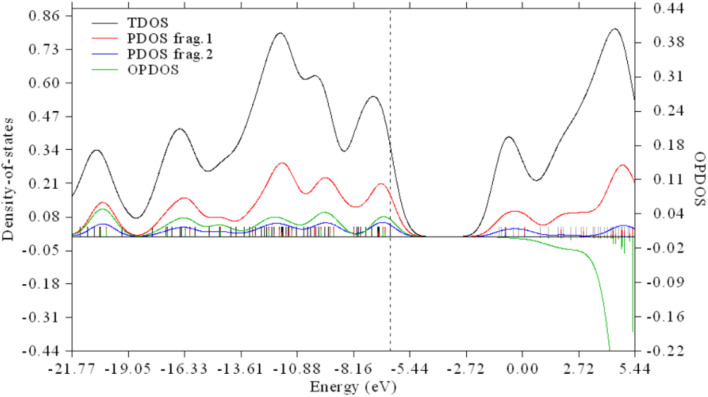
Partial density of states analysis of MO, BA and MO-BA (frag.1 is BA and frag.2 is MO).

The MO-BA co-crystal was screened for partial density of states (PDOS) analysis to gain information on its electronic structure and interactive forces.^48,49^ MO-BA showed peaks that overlap in the valence and conduction band regions, as indicated in Fig. 6. The plots show that the molecular orbitals of MO and BA coupled strongly to form the MO-BA co-crystal successfully. The PDOS observations are in agreement with the HOMO–LUMO gap.

**Fig. 7:**
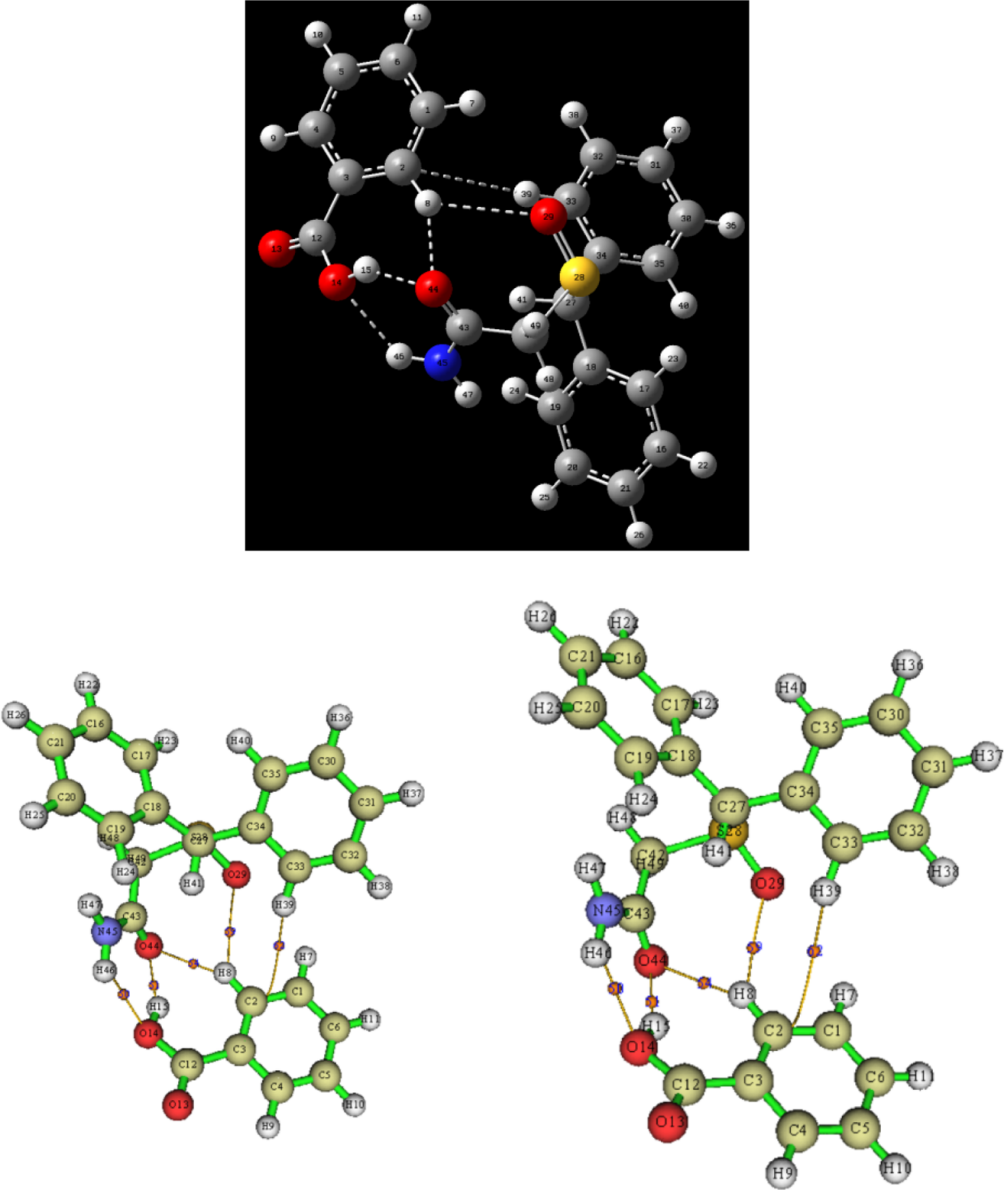
QTAIM molecular graphs of MO-BA showing the NCIs (intermolecular).

The QTAIM and NCI-RDG study was performed to understand the type and strength of intermolecular forces that are responsible for the stability of the MO-BA co-crystal. Bond critical points (BCPs) represent the bonding interactions between a pair of nuclei through parameters including the Laplacian of the density, total electron density and electron energy density.^50,51^ The topological molecular graphs of MO-BA show that it exhibits five BCPs, which are BCP 50, BCP 51, BCP 54 BCP 59 and BCP 62, found along the bond paths O(14)⋯H(46), O(44)⋯H(15), O(44)⋯H(8), O(29)⋯H(8), C(2)⋯H(39), respectively. The type and strength of the intermolecular forces can be predicted from the topological parameters. The interactions would be hydrogen bonding if *ρ*(*r*) is ∼10^−2^ a.u., ∇^2^*ρ*(*r*) > 0, and *H*(*r*) < 0. The interactions would be weak van der Waals if *ρ*(*r*) is ∼10^−3^ a.u., ∇^2^*ρ*(*r*) is greater than 0, and *H*(*r*) is also greater than 0. The BCP 50 and BCP 51 have ∇^2^*ρ*(*r*) > 0 and *H*(*r*) < 0 (shown in Table 2), which confirms that strong hydrogen-bonding interactions are present in the MO-BA co-crystal that are responsible for its stability, and other BCPs showed van der Waals interactions (Fig. 7).

**Table 2:** Laplacian of the electron density (∇^2^*ρ* a.u.), electron energy density (*H*(*r*) a.u.) and total electron density (*ρ* a.u.) of MO-BA

Co-crystal (NCD)	*E*_form_ (eV)	Bond critical points (BCP)	*ρ*(*r*) (a.u.)	∇^2^*ρ*(*r*) (a.u.)	*H*(*r*) (a.u.)
MO-BA	−0.71	BCP50, H(46)⋯O(14)	0.016	0.054	−0.00045
		BCP51, O(44)⋯H(15)	0.034	0.104	−0.00070
		BCP54, O(44)⋯H(8)	0.012	0.042	0.00072
		BCP59, H(8)⋯O(29)	0.005	0.021	0.00099
		BCP62, H(39)⋯C(2)	0.002	0.006	0.00035

**Fig. 8:**
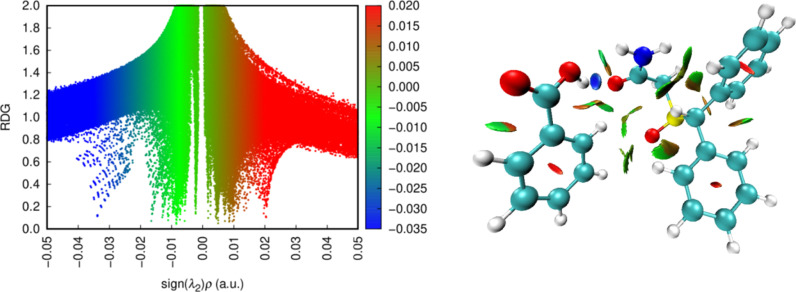
RDG isosurfaces of MO-BA and their corresponding NCI plots.

Reduced density gradient (RDG) analysis was performed to further verify the NCIs between MO and BA. The colored regions (blue, green and red) shown in Fig. 8 represent hydrogen bonding, van der Waals forces and repulsive steric interactions, respectively.^52^ The blue blobs between (MO) C=O⋯H–O–C=O (BA) and (MO) N–H⋯OH–C=O (BA) indicate hydrogen bonding between the carboxyl group of BA and the heteroatoms of modafinil. The green, broad band represents the presence of auxiliary weak van der Waals interactions such as π–π stacking in MO-BA. The NCI scatter graphs of RDG *versus* “sign6 (*λ*_2_) *p*” of MO-BA are given in Fig. 8. The NCIs can be distinguished from the values of “sign6 (*λ*_2_) *p*”. Their negative values represent attractive interactions, while the positive values illustrate repulsive interactions. The blue spike on the left near −0.04 and −0.03 corresponds to strong hydrogen bonding. The red spike near 0.02 represents a weak van der Waals interaction. The scatter NCI graphs support the previous finding of the RDG analysis.^53^

### 3.6 Solubility study and HPLC analysis

The solubilities of raw MO and synthesized MO-BA were determined in distilled water. The solubility values of MO and MO-BA were determined to be 0.52 ± 0.015 and 4.13 ± 0.012 mg mL^−1^, respectively, which indicated that the solubility of MO-BA has improved significantly compared to raw MO. The enhanced solubility of MO-BA was attributed to the co-former benzoic acid. Similarly, the molar ratio of MO in the MO-BA co-crystal was determined by the validated HPLC method. HPLC analysis of the co-crystal revealed that the concentration of MO was 65–67%, which is close to the theoretical value of 69%, thus confirming their 1 : 1 ratio. Hence, it is suggested that the MO-BA co-crystal is effectively designed in an equimolar ratio.

### 3.7 *In vitro* dissolution study

**Fig. 9:**
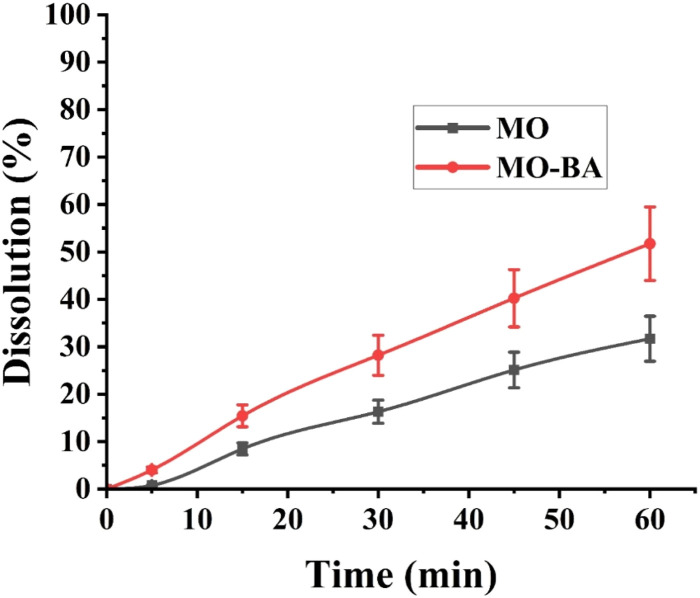
Percentage dissolution of MO and MO-BA in buffer solution of pH 6.8.

**Fig. 10:**
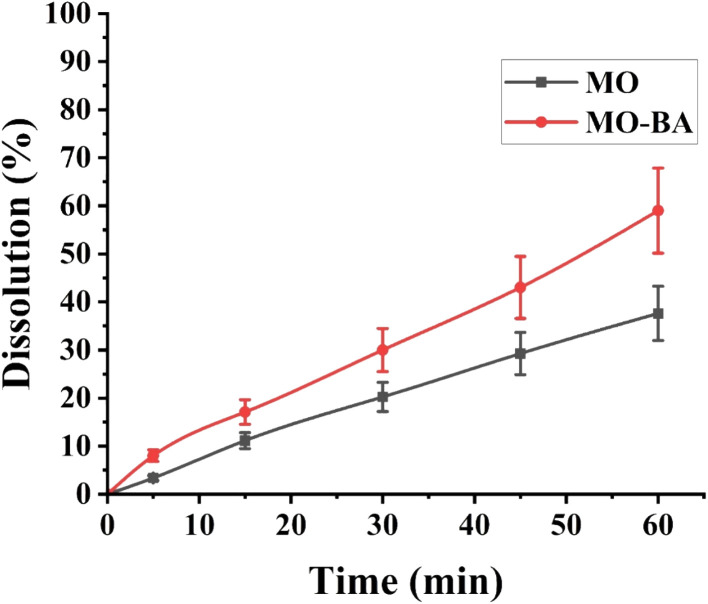
Percentage dissolution of MO and MO-BA in acidic media (HCl) of pH 1.3.

The *in vitro* dissolution potential of raw MO and its MO-BA co-crystal was performed in HCl media of pH 1.3 and in phosphate buffer at pH 6.8. The percentage dissolutions of MO and MO-BA in HCl media of pH 1.3 and pH 6.8 buffer solution are given in Fig. 9 and 10, respectively. The results obtained indicate that the MO-BA co-crystal exhibited a high dissolution percentage of 51.75% ± 1.55% in buffer solution of pH 6.8 and 59% ± 1.20% in HCl media of pH 1.3 as compared to raw modafinil with 31.42% ± 1.48% and 37.61% ± 1.85%, respectively. The results were determined 60 minutes after the introduction of the samples to the apparatus. The better dissolution rate of the MO-BA co-crystal in comparison to raw modafinil is due to the different sizes, crystal design and bulk form.

### 3.8 *In vivo* antidepressant activity

#### 3.8.1 Forced swim test

**Fig. 11:**
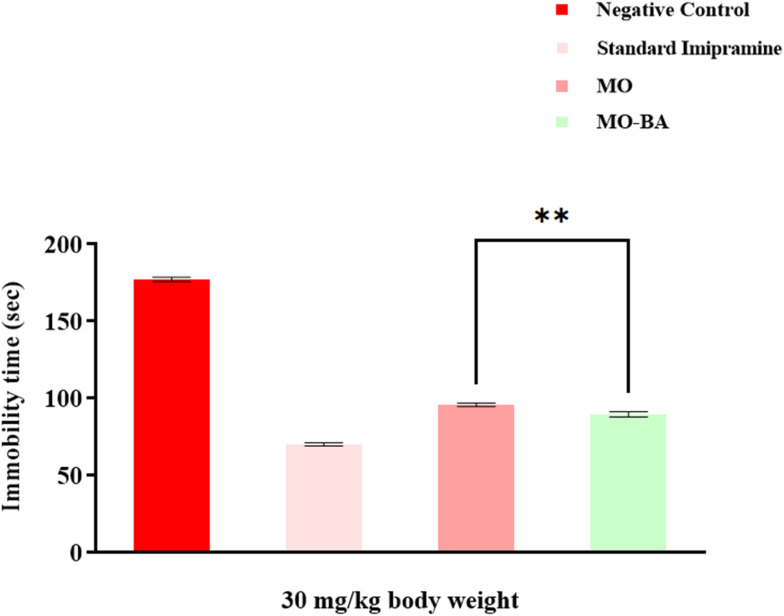
Forced swim tests of MO and MO-BA. (Data are expressed as mean ± SEM with *n* = 8. One-way ANOVA and Dunnett's *post hoc* test were used to determine the statistical significance. *P < 0.05, **P < 0.01, and ***P < 0.001 were considered significant when compared with the control group).

The forced swim test (FST) is the most useful model for the determination of the anti-depressant potential of new agents. The anti-depressant potentials of raw MO and MO-BA were assessed by using this model. FST results (Fig. 11) displayed that free MO produced an immobility time of 95.83 ± 1.13 s at a dose of 30 mg kg^−1^. Conversely, MO-BA induced an immobility response of 89.66 ± 1.78 s at a dose of 30 mg kg^−1^. The result was statistically significant, with *P* < 0.01 when MO and MO-BA were compared. The negative control group, to which only vehicle was administered, triggered an immobility response of 177.16 ± 1.49 s, while the imipramine standard group produced an immobility time of 70.16 ± 1.10 seconds.

#### 3.8.2 Tail-suspension test

**Fig. 12:**
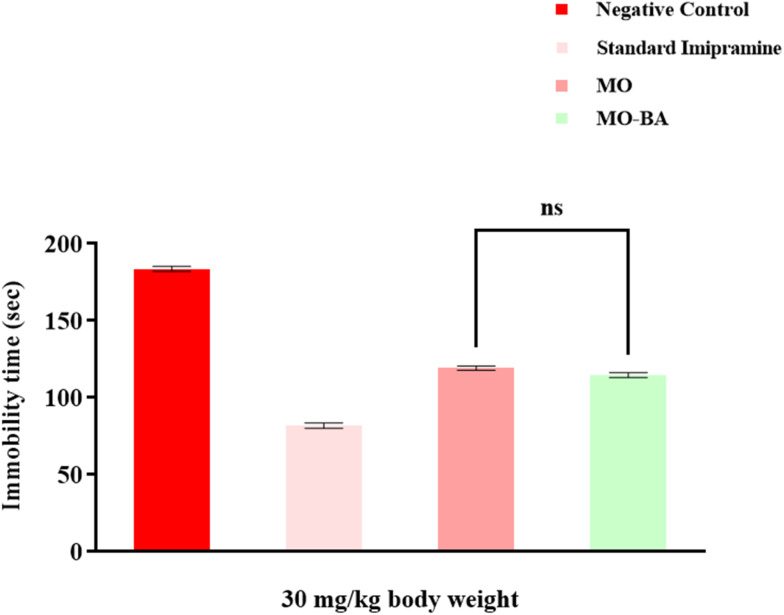
Tail-suspension tests of MO and MO-BA. (Data are expressed as mean ± SEM with *n* = 8. One-way ANOVA and Dunnett's *post hoc* test were used to determine the statistical significance. *P < 0.05, **P < 0.01, and ***P < 0.001 are considered significant when compared with the control group).

The tail-suspension test (TST) is typically used as a model to study the anti-depressant potential of new agents. Thus, this model was applied to assess the anti-depressant potential of raw MO and the MO-BA co-crystal. Free MO with a dose of 30 mg kg^−1^ instigated an immobility response of 119.16 ± 1.42 s, whereas MO-BA produced an immobility response of 114.66 ± 1.58 s with a dose of 30 mg kg^−1^. The negative control, to which only vehicle was administered, exhibited an immobility response of 183.66 ± 1.64 s, while the standard imipramine produced an immobility time of 81.83 ± 1.74 s. The results were found to be statistically non-significant when MO and MO-BA were compared (Fig. 12).

## 4 Conclusions

The pharmacokinetic properties of active pharmaceutical ingredients (API) can be improved by non-covalent derivatization. In this study, the API modafinil (MO) was bridged with co-former (BA), and a new MO-BA co-crystal was successfully synthesized by the slow evaporation method. HPLC analysis revealed that MO and BA combine in a stoichiometric ratio (1 : 1) to form MO-BA. The presence of hydrogen bonding between MO and BA was indicated by FT-IR spectroscopy as broadening, attenuation and shifting of the –OH, –NH, –C=O and –S=O peaks. DSC and PXRD analyses confirmed the formation of MO-BA by revealing disparities in thermograms and diffractograms, respectively, of MO, BA and MO-BA. The negative value of the *E*_form_ of MO-BA revealed by DFT analysis confirmed that it is thermodynamically stable. The presence of hydrogen bonding and van der Waals interactions in MO-BA was further confirmed by QTAIM and NCI analyses. The *in vitro* dissolution study revealed that the MO-BA co-crystal exhibited better dissolution compared to raw MO. In the *in vivo* anti-depressant model, the MO-BA was statistically significant (*p* = 0.01) in the forced swim test, while statistically non-significant in the tail-suspension test.

## Ethical statement

All animal procedures were performed in accordance with the Guidelines for Care and Use of Laboratory Animals of Malakand University and approved by the Animal Ethics Committee of the Department of Pharmacy, UOM (Pharm/ethics/2025/234-90).

## Author contributions

All authors read and approved the manuscript. ZH, MNU and AK: idea; MZ and SWAS: first draft; ZH, MG and AA: literature search; ZH, AK and AA: revision of the manuscript; MZ, SWAS and AK: manuscript write-up and literature search; and ZH, AK and MZ: software.

## Conflicts of interest

The authors of this article have no conflicts of interest.

## Data Availability

All data are presented in this manuscript. There are no associated data in any repository.
